# Supplementary material for: LIGHTHOUSE illuminates therapeutics for a variety of diseases including COVID-19
Source: iScience. 2022 Oct 10;25(11):105314. doi: 10.1016/j.isci.2022.105314 (PMC9549714; doi:10.1016/j.isci.2022.105314)
Supplement: Document S1. Figures S1–S13, Tables S1–S4 and S7–S9 [file mmc1.pdf]

iScience, Volume *25*

Supplemental information

LIGHTHOUSE illuminates therapeutics

for a variety of diseases including COVID-19

Hideyuki Shimizu, Manabu Kodama, Masaki Matsumoto, Yasuko Orba, Michihito Sasaki, Akihiko Sato, Hirofumi Sawa, and Keiichi I. Nakayama

SUPPLEMENTAL INFORMATION

LIGHTHOUSE illuminates therapeutics for a variety of diseases including COVID-19

Hideyuki Shimizu, Manabu Kodama, Masaki Matsumoto, Yasuko Orba, Michihito Sasaki, Akihiko Sato, Hirofumi Sawa, and Keiichi I. Nakayama

SUPPLEMENTAL TABLE TITLES

SUPPLEMENTAL TABLES 1–4 AND 7–9 (SUPPLEMENTAL TABLES 5 and 6 ARE PROVIDED SEPARATELY IN CSV FORMAT)

SUPPLEMENTAL FIGURE LEGENDS

SUPPLEMENTAL FIGURES 1–13

SUPPLEMENTAL TABLE TITLES

Table S1. Stratified sampling of the STITCH database, Related to Figure 1.

Table S2. Final performance of LIGHTHOUSE for confidence scores with test data, Related to Figure 1.

Table S3. Final performance of LIGHTHOUSE for interaction scores with test data, Related to Figure 1.

Table S4. Comparison of model performance for LIGHTHOUSE and other state-of-the-art methods, Related to Figure 2.

Table S5. Confidence and interaction scores for WHO essential drugs and their target proteins, Related to Figure 2. (PROVIDED SEPARATELY IN CSV FORMAT)

Table S6. Confidence scores for statins, Related to Figure 6. (PROVIDED SEPARATELY IN CSV FORMAT)

Table S7. Median effective concentration (EC_{50}) values of ethoxzolamide for Vero-TMPRSS2 cells challenged with various SARS-CoV-2 strains, Related to Figure 7.

Table S8. Differences in confidence and interaction scores for SARS-CoV-2 proteins and either ethoxzolamide or acetazolamide, Related to Figure 7.

Table S9. Median effective concentration (EC_{50}), median cytotoxic concentration (CC_{50}), and selectivity index of 12 approved drugs for Vero-TMPRSS2 cells challenged with various SARS-CoV-2 strains, Related to Figure 7.

Score	STITCH original	Stratified sampling
0.0 ~ 0.2	5,310,242	140,000
0.2 ~ 0.3	5,237,589	140,000
0.3 ~ 0.4	1,769,008	140,000
0.4 ~ 0.5	507,870	140,000
0.5 ~ 0.6	264,566	140,000
0.6 ~ 0.7	200,401	140,000
0.7 ~ 0.8	161,656	140,000
0.8 ~ 0.9	143,333	140,000
0.9 ~ 1.0	169,391	140,000
	13,764,056	1,260,000

Shimizu et al., Table S1

Performances for confidence score

	MPNN_CNN	MPNN_AAC	MPNN_Transformer
MSE (test data)	0.0222	0.0207	0.0195
AUROC (test data)	0.8082	0.8212	0.8280

Shimizu et al., Table S2

Perfomances for interaction score

	MPNN_CNN	MPNN_AAC	MPNN_Transformer
MSE (test data)	0.5820	0.5764	0.5795
AUROC (test data)	0.8432	0.8437	0.8428

Shimizu et al., Table S3

Perfomances for the dataset Tsubaki et al. provided
(https://github.com/masashitsubaki/CPI_prediction/tree/master/dataset)

	Graph-based deep learning methods	
	Tsubaki et al.	LIGHTHOUSE
**Human**		
AUROC	0.970	**0.991**
F1	0.920	**0.957**
**C. elegans**		
AUROC	0.978	**0.989**
F1	0.933	**0.952**

Shimizu et al., Table S4

(SUPPLEMENTARY TABLE 5 AND 6 ARE PROVIDED SEPARATELY IN CSV FORMAT)

EC50 (µM) of Ethoxzolamide (mean ± SD)	
SARS-CoV-2 Wuhan	3.71 ± 0.709
SARS-CoV-2 U.K.	3.14 ± 0.429
SARS-CoV-2 South Africa	14.5 ± 0.689
SARS-CoV-2 Brazil	18.5 ± 1.74
SARS-CoV-2 India	6.18 ± 0.738

Shimizu et al., Table S7

Top 0.3% entries in UniProt sorted by delta confidence

UniProt ID	UniProt ID	Annotated function	⊿ confidence	⊿ interaction
A0A7T0FY88	S	Spike_S1_RBD	+0.135	+0.356
A0A8B1L1L6	ORF7a	Host-virus interaction	+0.131	+0.820
A0A8B1IX79	ORF7a	Host-virus interaction	+0.131	+0.820
A0A8B6RWD9	ORF7a	Host-virus interaction	+0.131	+0.820
A0A8B1JCN7	ORF7a	Host-virus interaction	+0.131	+0.820
A0A8B1JSC3	S	Spike_S1_RBD	+0.127	+0.317
A0A8B1JVW1	S	Spike_S1_RBD	+0.125	+0.287
A0A8B6RA66	S	Spike_S1_RBD	+0.124	+0.234
A0A883GQB7	S	Spike_S1_RBD	+0.124	+0.253
A0A8B6RFK5	ORF7a	Host-virus interaction	+0.123	+0.605
A0A8B6REM7	S	Spike_S1_RBD	+0.123	+0.229
A0A8B6RNI3	S	Spike_S1_RBD	+0.122	+0.349
A0A8B1JGU2	S	Spike_S1_RBD	+0.122	+0.305
A0A8B1KVV0	ORF8	-	+0.121	+1.20

Shimizu et al., Table S8

		VeroE6T cells		
Supplier	Catalog number	EC50 (nM)	CC50 (nM)	Selectivity Index
**Sigma-Aldrich**	**333328-1G**	**1698**	**62192**	**37**
TCI	A2598	>100000		
**Sigma-Aldrich**	**D4628-1G**	**5695**	**12609**	**2.2**
Sigma-Aldrich	N5535-100G	>100000		
Sigma-Aldrich	D150959-5G	>100000		
TCI	A2476	>100000		
**TCI**	**R0064**	**9579**	**41705**	**4.4**
TCI	G0392	>100000		
Sigma-Aldrich	46959-U	>100000		
Sigma-Aldrich	T3125-25MG	>100000		
Sigma-Aldrich	C7869-1MG	>100000		
Funakoshi	CS-0153	>100000		

Shimizu et al., Table S9

SUPPLEMENTAL FIGURE LEGENDS

Figure S1. Training process for determination of the confidence score by LIGHTHOUSE, Related to Figure 1

(A) More than 1 million chemical–(human) protein interactions (CPIs) were extracted from the STITCH database (http://stitch.embl.de) and were divided into a training set (80%), a validation set (10%), and a test set (10%). The registered confidence scores ranged from 0.15 to 1 and were calculated on the basis of experimental data, evolutionary relations, co-presentation in PubMed abstracts, and other factors. After one epoch of training, the validation data were applied. After every 10 epochs, the current loss (validation data set) was compared with the loss of 10 epochs ago to determine whether additional training was necessary. Finally, the test data were applied to evaluate model performance.
(B and C) MSE (B) and AUROC (C) for the validation data set with MPNN_CNN, which uses MPNN as the chemical encoder and CNN as the protein encoder.
(D and E) MSE (D) and AUROC (E) for the validation data set with MPNN_AAC, which uses MPNN as the chemical encoder and AAC as the protein encoder.
(F and G) MSE (F) and AUROC (G) for the validation data set with MPNN_Transformer, which uses MPNN as the chemical encoder and Transformer as the protein encoder.

Figure S2. Training process for determination of the interaction score by LIGHTHOUSE, Related to Figure 1

(A) More than 1 million reported IC_{50} values were extracted from the BindingDB database (https://www.bindingdb.org/bind/index.jsp) and were split into training (80%), validation (10%), and test (10%) data sets. Given that these values range widely, they were log-transformed. After one epoch of training, the validation data were applied. After every 10 epochs, the current loss (validation data set) was compared with the loss of 10 epochs ago to determine whether further training was necessary. The test data were finally examined to evaluate model performance.
(B and C) MSE (B) and AUROC (C) for the validation data set with MPNN_CNN, which uses MPNN as the chemical encoder and CNN as the protein encoder.
(D and E) MSE (D) and AUROC (E) for the validation data set with MPNN_AAC, which uses MPNN as the chemical encoder and AAC as the protein encoder.
(F and G) MSE (F) and AUROC (G) for the validation data set with MPNN_Transformer, which uses MPNN as the chemical encoder and Transformer as the protein encoder.

Figure S3. LIGHTHOUSE outperforms other state-of-the-art methods with

DUD-E data, Related to Figure 2

(A) DUD-E data were randomly split into training (72 proteins) and test (30 proteins) data. The LIGHTHOUSE architecture was trained with the DUD-E training data set, and its performance was then evaluated with the test data set.

(B) ROC curve for LIGHTHOUSE with the DUD-E test data.

(C) Comparison of the performance of LIGHTHOUSE with that of other state-of-the-art methods including a graph-based deep learning model (Tsubaki et al., 2019). Each model was trained with the same DUD-E training data, and the AUROC values for the DUD-E test data were compared.

Figure S4. LIGHTHOUSE score distributions for human proteins and representative small molecules, Related to Figure 2

Confidence and interaction score distributions are shown for all human proteins and either ATP (A), sorafenib (B), or sunitinib (C).

Figure S5. Most targets of sorafenib possess high confidence and interaction scores, Related to Figure 2

(A) Score distribution for all human proteins and sorafenib. Nine out of 10 known sorafenib targets are enriched in the upper right corner (black box, confidence score of >0.70 and interaction score of >7.0).

(B) The highest score region (green box in left panel, confidence score of >0.75 and interaction score of >7.5) contains 25 proteins (right panel), of which 10 proteins are known (8 proteins, shown in red) or putative (2 kinases, shown in blue) targets of sorafenib.

Figure S6. LIGHTHOUSE precisely predicts IC_{50} values, Related to Figure 3

Interaction scores predicted by LIGHTHOUSE and actual IC_{50} values are highly correlated (Spearman correlation $r = 0.886$) for BindingDB test data.

Figure S7. Prediction of potential PPAT inhibitors, Related to Figure 3

The amino acid sequence of PPAT and the SMILE representation of each compound in the ZINC database were entered into LIGHTHOUSE for virtual screening.

Figure S8. Combining confidence and interaction scores helps to predict drug-protein interactions, Related to Figures 2 and 3

(A) A linear combination of confidence and interaction scores was optimized, and the combined score was defined as confidence score + 0.075*interaction score. Two data sets were generated from STITCH: a “Positive” data set consisting of CPIs with high

scores (>0.9), and a “Negative” data set consisting of the same CPIs but with the amino acid sequences of the proteins reversed. See also Figure 2A and STAR★METHODS.
(B) ROC curve showing that the combined score further discriminates the two data sets (AUC of 0.867, compared with the value of 0.838 in Figure 2B).

Figure S9. Ethoxzolamide rescues cells infected with SARS-CoV-2, Related to Figure 7
Vero-TMPRSS2 cells challenged with alpha (A), beta (B), or gamma (C) strains of SARS-CoV-2 were cultured in the presence of various concentrations of ethoxzolamide for 3 days and then subjected to the MTT assay of cell viability. Nonchallenged cells were examined as a control. Data are means ± SD for three independent experiments.

Figure S10. Ethoxzolamide reduces SARS-CoV-2 virus load, Related to Figure 7
Vero-TMPRSS2 cells challenged with alpha (A), beta (B), or gamma (C) strains of SARS-CoV-2 were cultured in the presence of various concentrations of ethoxzolamide for 3 days, after which virus load in the culture supernatants was determined. Data are from four independent experiments, with the graph line connecting mean values. $TCID_{50}$, median tissue culture infectious dose; N.D., not detected.

Figure S11. Comparison between ethoxzolamide and acetazolamide with regard to targeting of SARS-CoV-2, Related to Figure 7
LIGHTHOUSE scores were calculated for all amino acid sequences associated with SARS-CoV-2 (https://www.uniprot.org/taxonomy/2697049) and for either ethoxzolamide or acetazolamide. The differences in the confidence and interaction scores (scores for ethoxzolamide minus scores for acetazolamide) were designated delta confidence and delta interaction scores and plotted. The most prominent difference between the two drugs was that a series of virus proteins involved in interaction with host cells scored higher for ethoxzolamide (pink box). See also Table S8.

Figure S12. Molecular docking simulation suggests that ethoxzolamide binds to the interface between the SARS-CoV-2 spike protein and ACE2, Related to Figure 7
The docking simulation shows that ethoxzolamide may inhibit the interaction between the virus S protein (green) and human ACE2 (gold).

Figure S13. Harnessing of transfer learning for automated lead compound optimization, Related to Figure 5

Inspired by the idea of reinforcement learning, LIGHTHOUSE could act as an "environment" for further refinement of lead compounds. A series of new virtual compounds is generated by the "Agent" and submitted to LIGHTHOUSE ("Environment"). Output scores for the target protein ("Reward") are then harnessed for the next generation of virtual compounds. Iteration of these cycles should allow the "Agent" to learn what are much improved compounds for the protein of interest.

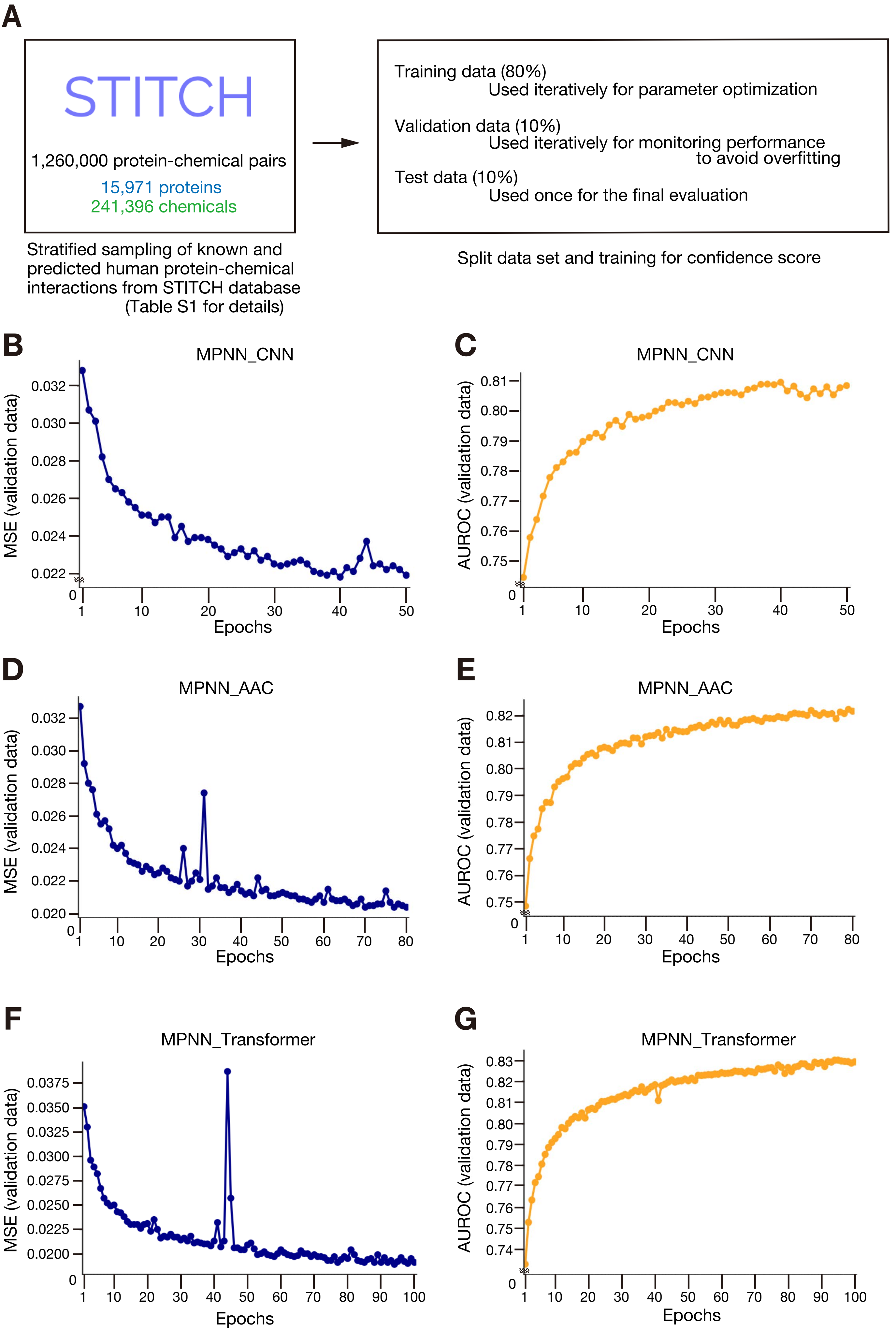

Shimizu et al., Figure S1

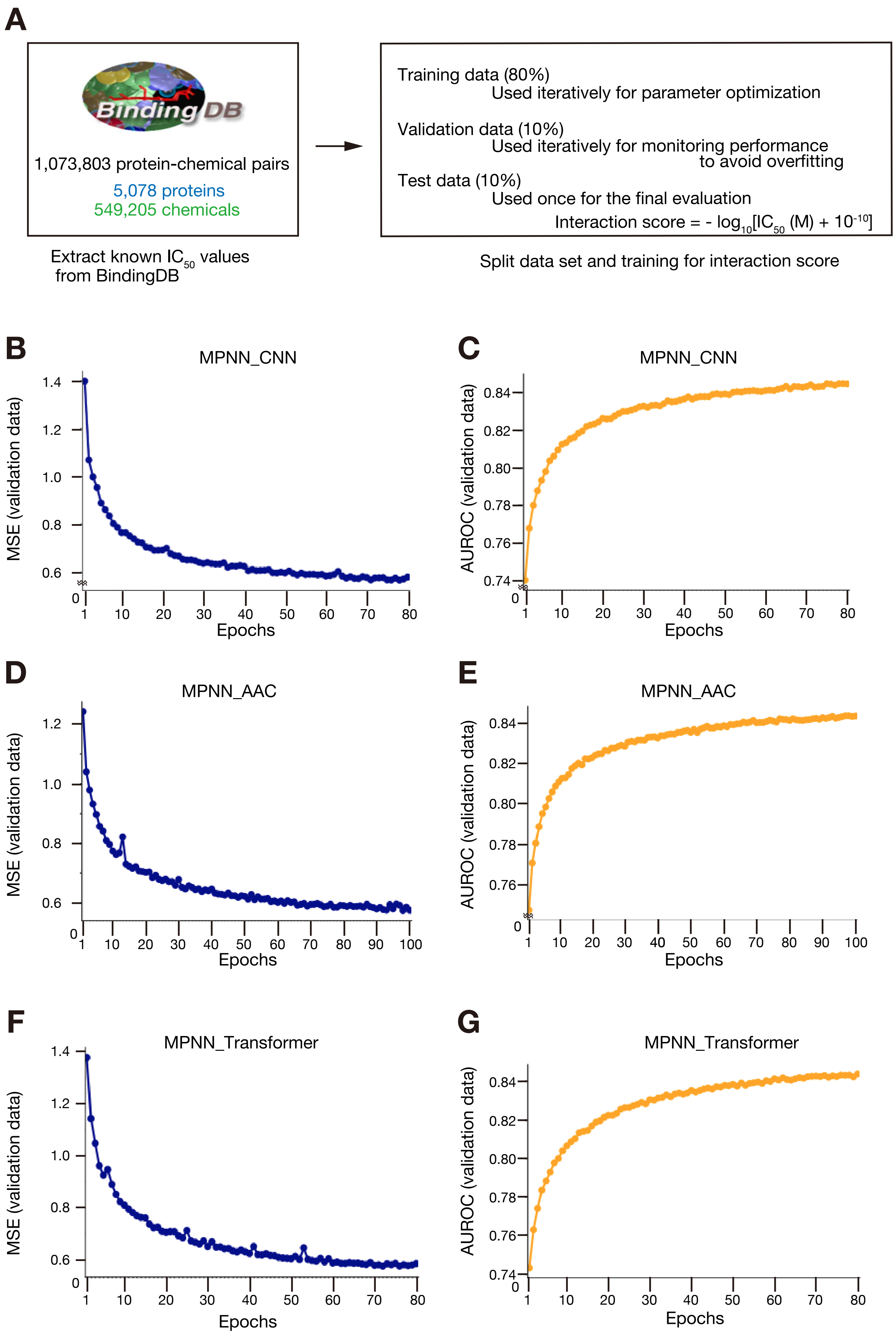

Shimizu et al., Figure S2

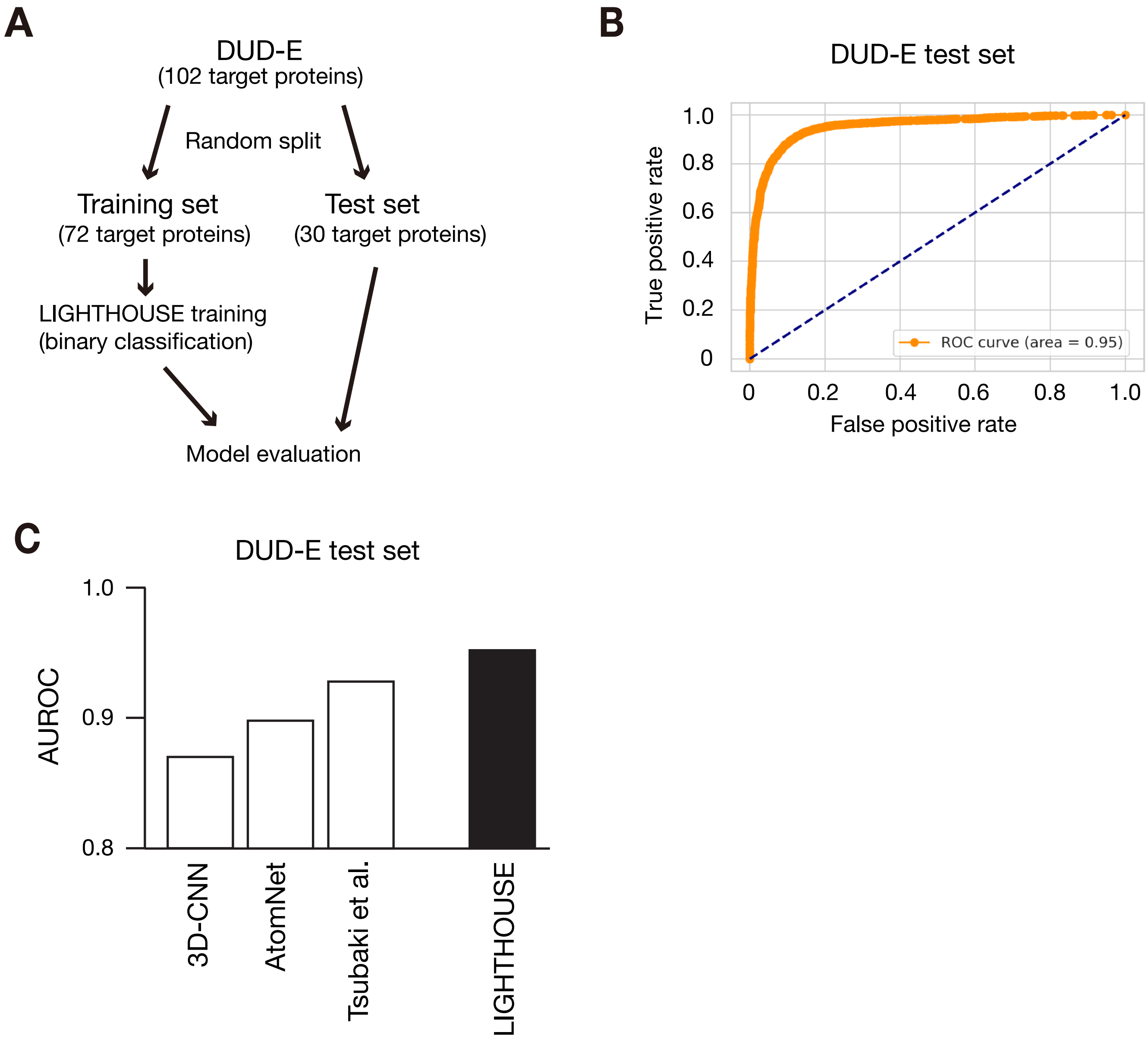

Shimizu et al., Figure S3

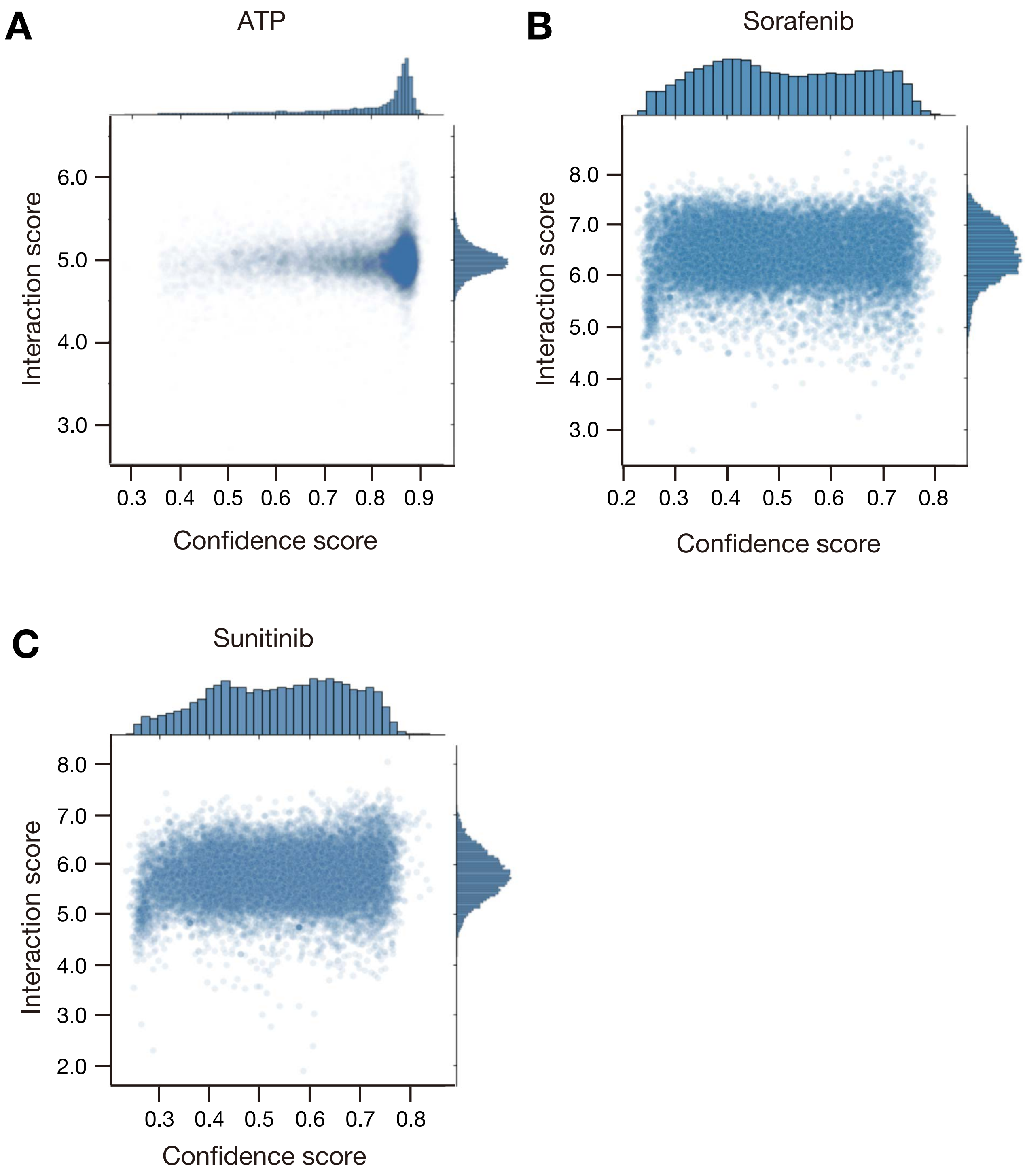

Shimizu et al., Figure S4

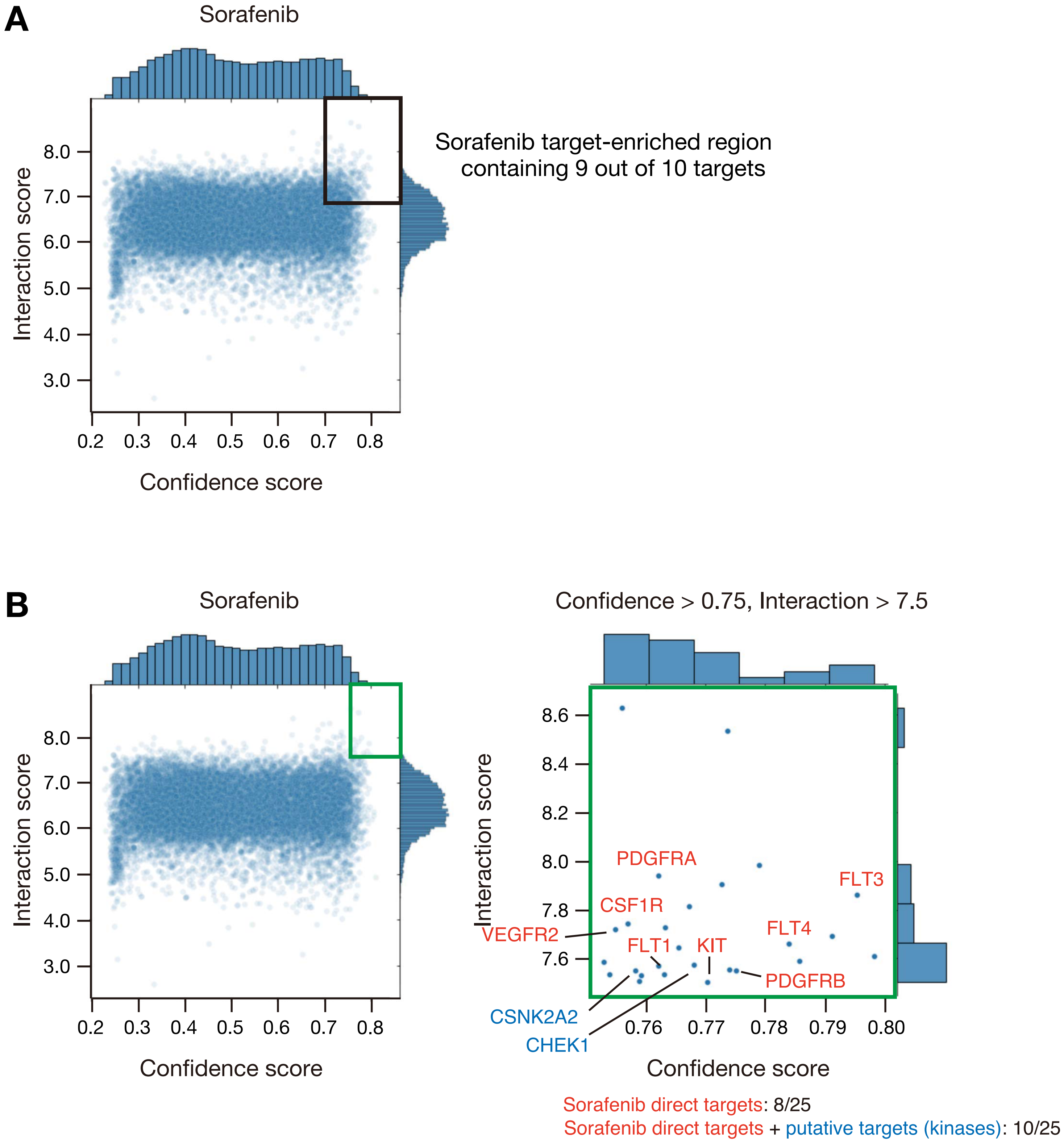

Shimizu et al., Figure S5

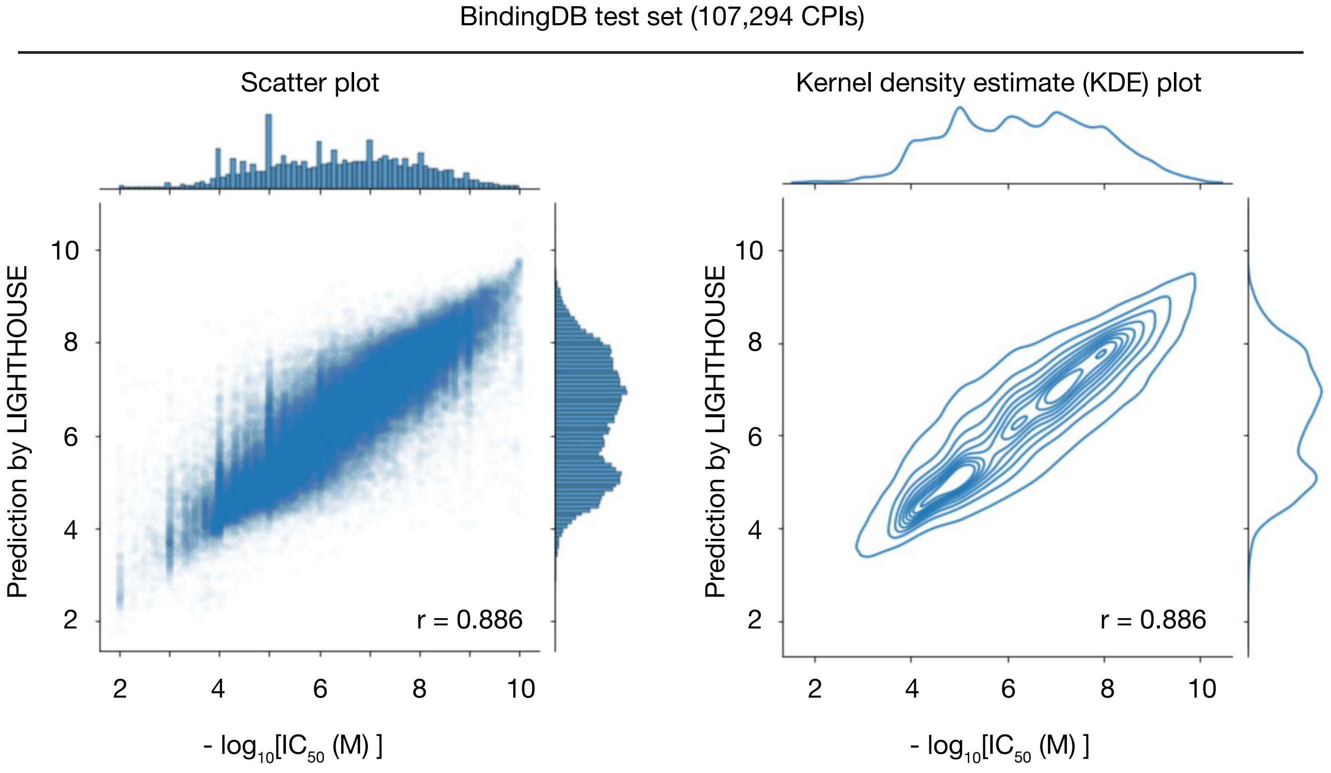

Shimizu et al., Figure S6

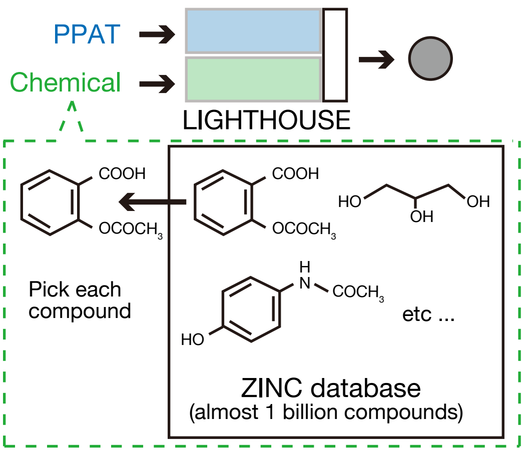

Shimizu et al., Figure S7

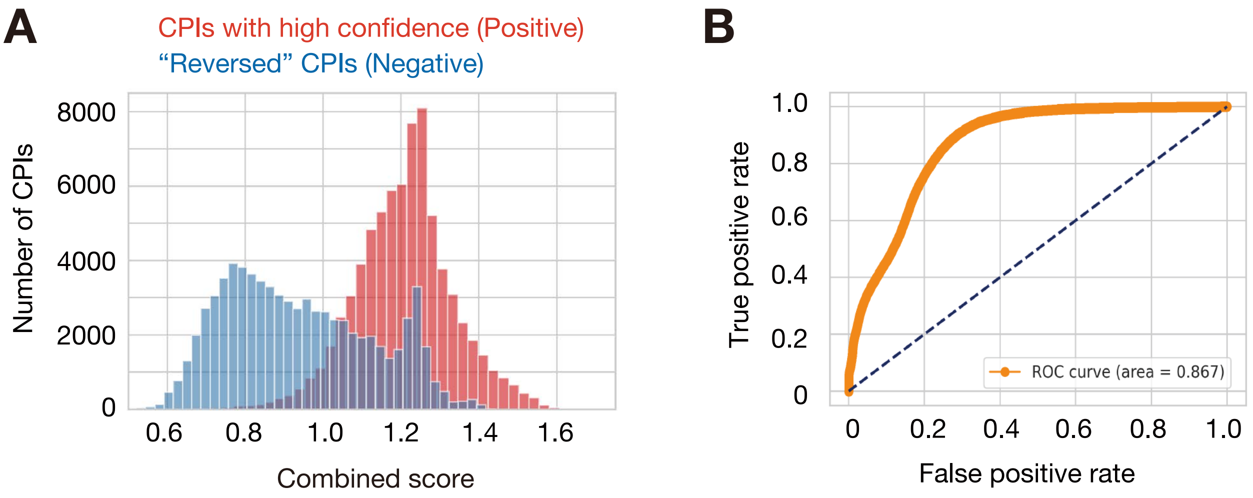

Shimizu et al., Figure S8

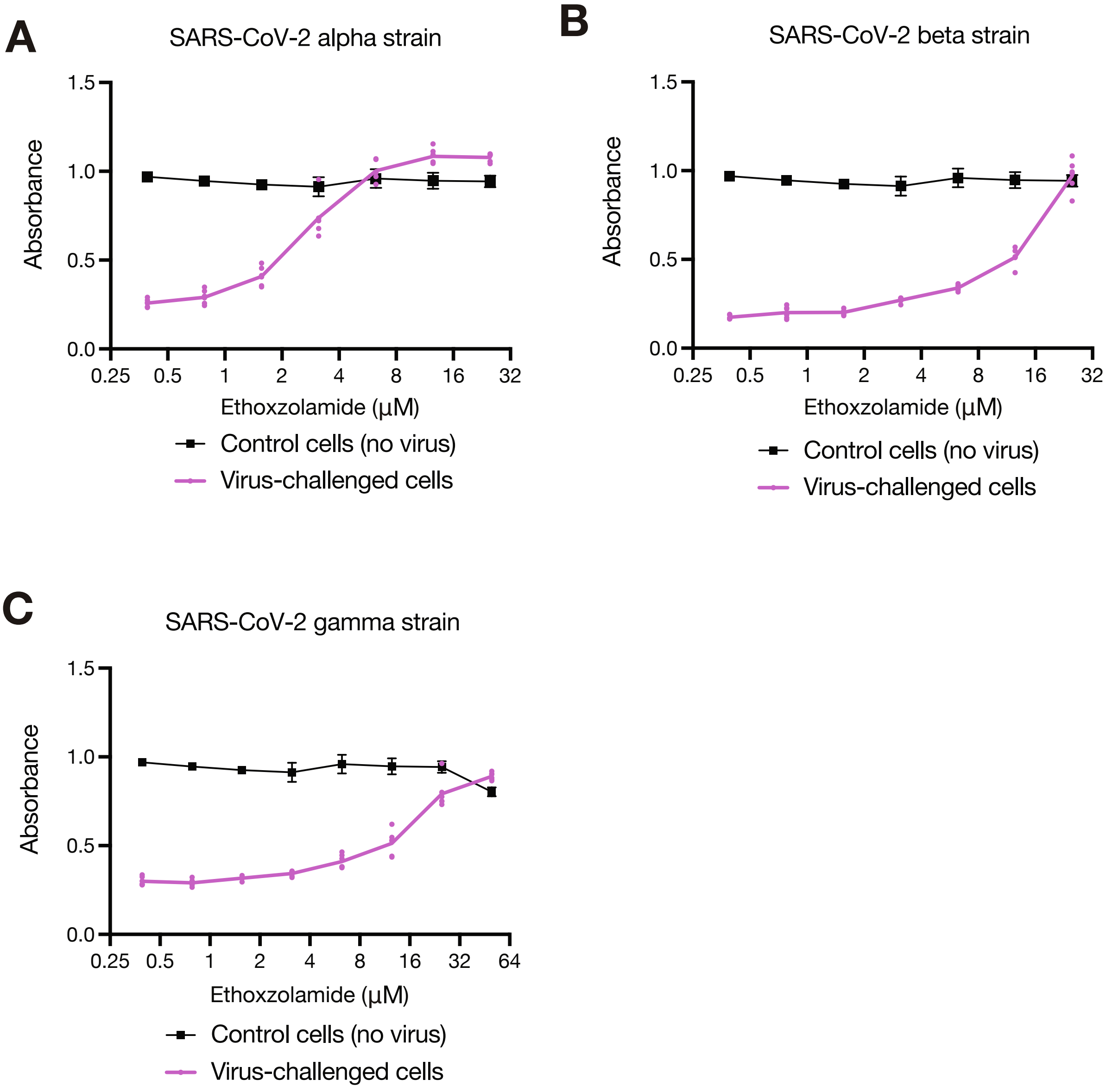

Shimizu et al., Figure S9

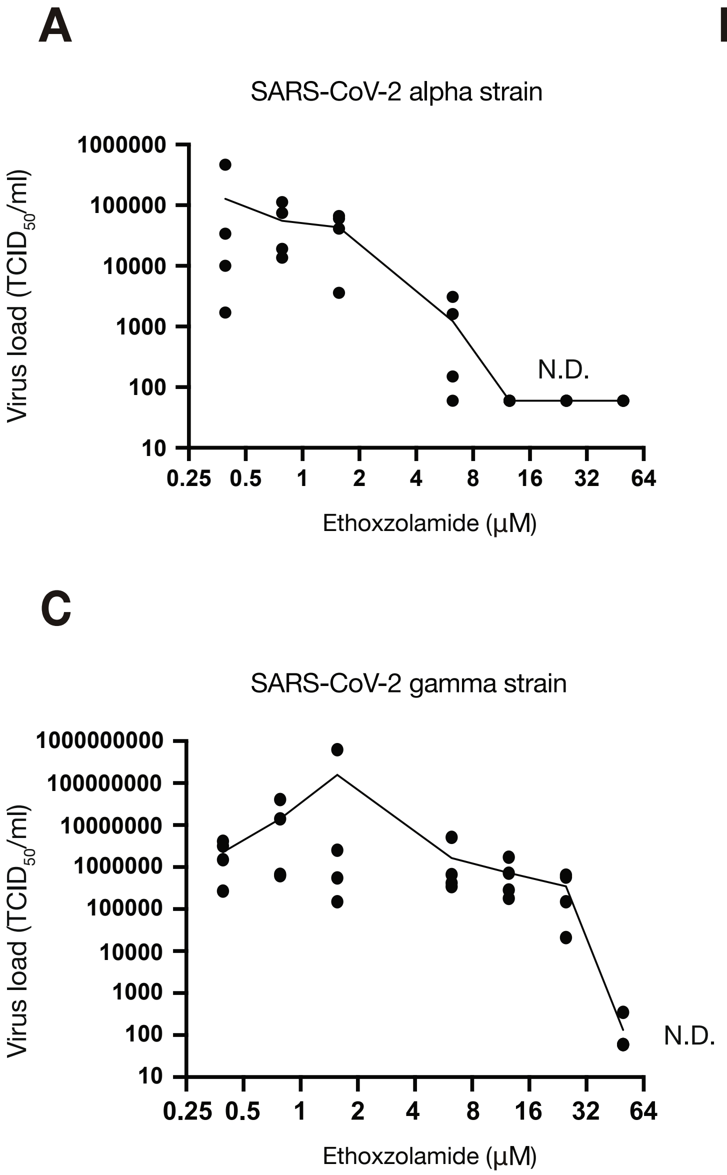

Shimizu et al., Figure S10

Severe acute respiratory syndrome coronavirus 2 (2019-nCoV) (SARS-CoV-2)

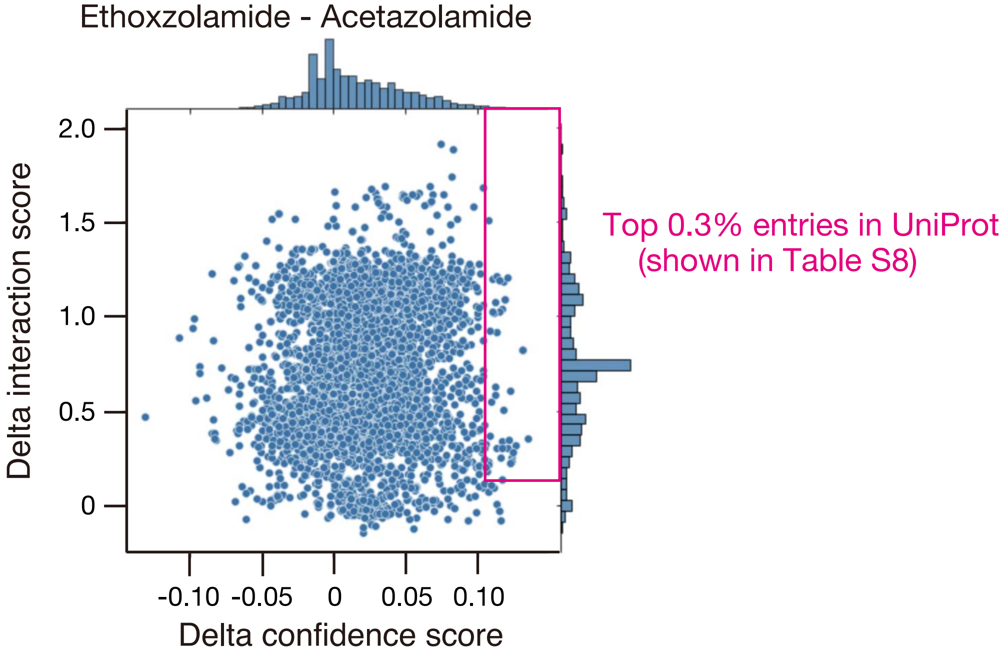

Shimizu et al., Figure S11

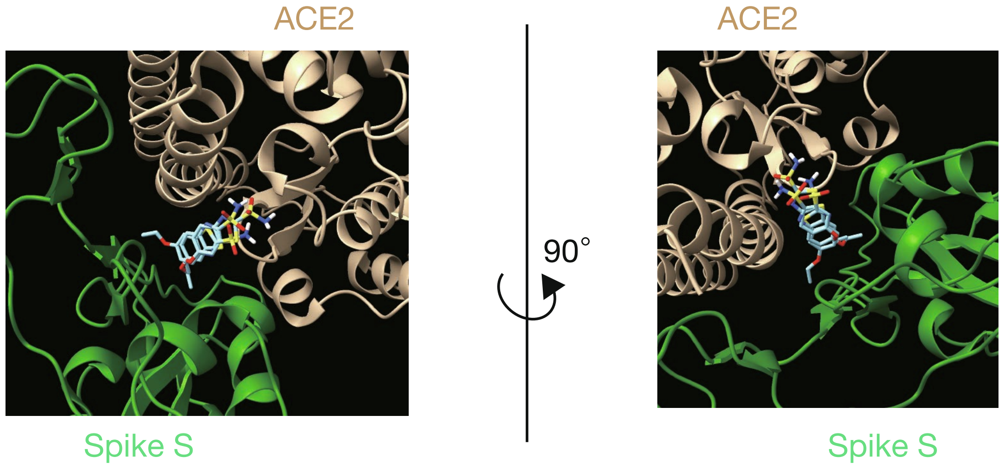

Shimizu et al., Figure S12

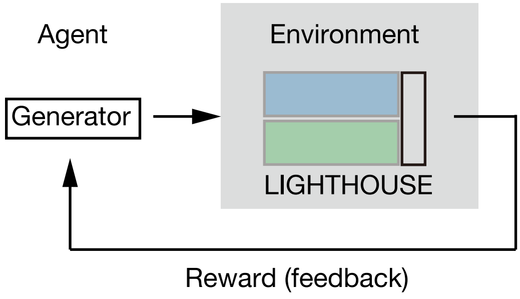

Shimizu et al., Figure S13
